# Cell-Based Small-Molecule Compound Screen Identifies Fenretinide as Potential Therapeutic for Translocation-Positive Rhabdomyosarcoma

**DOI:** 10.1371/journal.pone.0055072

**Published:** 2013-01-25

**Authors:** David Herrero Martín, Aleksandar Boro, Beat W. Schäfer

**Affiliations:** Department of Oncology and Children’s Research Center, University Children’s Hospital Zurich, Zurich, Switzerland; Ospedale Pediatrico Bambino Gesù, Italy

## Abstract

A subset of paediatric sarcomas are characterized by chromosomal translocations encoding specific oncogenic transcription factors. Such fusion proteins represent tumor specific therapeutic targets although so far it has not been possible to directly inhibit their activity by small-molecule compounds. In this study, we hypothesized that screening a small-molecule library might identify already existing drugs that are able to modulate the transcriptional activity of PAX3/FOXO1, the fusion protein specifically found in the pediatric tumor alveolar rhabdomyosarcoma (aRMS). Towards this end, we established a reporter cell line based on the well characterized PAX3/FOXO1 target gene AP2ß. A library enriched in mostly FDA approved drugs was screened using specific luciferase activity as read-out and normalized for cell viability. The most effective inhibitor identified from this screen was Fenretinide. Treatment with this compound resulted in down-regulation of PAX3/FOXO1 mRNA and protein levels as well as in reduced expression of several of its direct target genes, but not of wild-type FOXO1, in a dose- and time-dependent manner. Moreover, fenretinide induced reactive oxygen species and apoptosis as shown by caspase 9 and PARP cleavage and upregulated miR-9. Importantly, it demonstrated a significant anti-tumor effect *in vivo*. These results are similar to earlier reports for two other pediatric tumors, namely neuroblastoma and Ewing sarcoma, where fenretinide is under clinical development. Our results suggest that fenretinide might represent a novel treatment option also for translocation-positive rhabdomyosarcoma.

## Introduction

Prognosis of cancer patients can be strongly improved through optimization of therapeutic regimens. However, to increase survival rates it is necessary to identify and develop further alternative treatment strategies as well as to minimize treatment related side-effects, a matter of great importance for childhood cancer long-term survivors. In our studies, we use the pediatric tumor rhabdomyosarcoma (RMS) as a model system. RMS accounts for 5% to 8% of all pediatric malignancies and is the most common soft tissue sarcoma diagnosed in children [1]. Histopathologically, RMS is classified into two main subtypes, embryonal RMS (eRMS) and alveolar RMS (aRMS). eRMS represents the majority of RMS cases, ∼60%, and shows a more favorable prognosis [2]. aRMS is less frequent, more aggressive, appears at unfavourable locations, frequently presents with metastasis and displays resistance to conventional chemo- and radiotherapy [3]. The majority of aRMS is characterized by the specific chromosomal translocation t(2;13)(q35;q14) generating the chimeric transcription factor PAX3/FOXO1 [4].

The oncogenic role of PAX3/FOXO1 depends on deregulation of PAX3 target genes as well as alteration of gene expression patterns [5], [6]. Down-regulation or inhibition of PAX3/FOXO1 inhibits proliferation and induces apoptosis in aRMS cells [7], [8], [9], hence the aRMS tumor cells become dependent on fusion protein expression. PAX3/FOXO1 transcriptional activity is subject to modulation at different molecular levels such as e.g. post-translational modifications. Phosphorylation of several serine residues in a peptide located between the two DNA-binding domains of PAX3 is required for efficient target gene activation [10]. Furthermore, stability of the fusion protein itself is subject to regulation via the proteasome pathway [11], [12]. Thus, oncogenic fusions are necessary for maintaining the malignant phenotype of aRMS which spots them as being ideal targets for development of a directed therapeutic approach.

Dose intensification of conventional multimodal chemotherapeutic regimens confers small survival benefits for RMS patients with metastatic or recurrent disease [13], [14]. Unfortunately, there are no alternative treatment strategies so far that would target tumor cells more specifically and effectively [15]. Therefore, we aimed at identifying drugs capable to modulate the transcriptional activity of PAX3/FOXO1 irrespective of their mode of action. In this study, we used a small-molecule compound library coupled with a specific reporter assay to identify fenretinide as the most promising drug inhibiting PAX3/FOXO1 activity. Fenretinide (N-(4-hydroxyphenyl) retinamide) is a synthetic vitamin A analogue with chemopreventive [16] and known anti-tumoral activity [17]. It shows a wide range of effects in different carcinomas and importantly, is already under clinical development in several childhood malignancies [18]. Hence, our study identifies with aRMS yet another pediatric tumor that might be sensitive to fenretinide treatment.

## Materials and Methods

### Cell Lines and Reagents

aRMS cell lines Rh4 [19] and Rh41 [20] were kindly provided by Dr. P. Houghton (Nationwide Children’s Hospital, Columbus, Ohio) and RMS13, Rh30 (aRMS), RD (eRMS) and HEK 293 cells were obtained from American Type Culture Collection (LGC Promochem, Molsheim, France). Ruch2 (eRMS, botryoid subtype) was established in our laboratory [21]. ES cell lines, TC71 and A673, were obtained from American Type Culture Collection. All cells were maintained in high glucose DMEM (Bioconcept, Allschwil, Switzerland) supplemented with 10% fetal calf serum, 2 mM L-glutamine, 100 U/ml penicillin and 100 mg/ml streptomycin, in 5% CO_2_ at 37°C. The small-compound library (LOPAC 1280), fenretinide, phorbol 12-myristate 13-acetate (TPA) and L-ascorbic acid were purchased from Sigma-Aldrich (Buchs, Switzerland).

### Drug-screening

PAX3/FOXO1 luciferase reporter was based on the well characterized AP2beta promoter and generated as previously described in the pGL3 vector [10], [11]. The pGL3 enhancer vector was used as control. Vectors were electroporated individually into Rh4 cells using the AMAXA system (Lonza, Cologne, Germany) (Program O17, buffer R). Selection of stably transfected cells as pool was performed with 1 mg/ml G-418 sulfate (Promega, Wallisellen, Switzerland). 5×10^3^ Rh4-AP2β-LF or SV40-LF cells were plated into 96 well plates and treated 24 hours later with the small-molecule compounds at a final concentration of 5 µM during 24 hours. Afterwards, luciferase activity was measured (Luciferase Assay System, Promega) according to manufactureŕs protocol in parallel with measuring proliferation rates (WST-1) (Roche, Basel, Switzerland) in the same wells to correct for cell viability.

### Proliferation Assay

Twenty-four hours after seeding in 96-well plates, cells (5×10^3^ per well) were treated with increasing concentrations of fenretinide (Sigma-Aldrich) in a final volume of 100 µL medium including 10% serum for 72 hours. WST-1 assay (Roche) was then performed according to the manufacturer’s instructions. For each concentration, the percentage of viable cells compared with control cells was plotted against the logarithm of drug concentrations. IC50 values were then calculated by nonlinear regression curve fitting using GraphPad Prism software (GraphPad Software Inc., San Diego, CA, USA).

### Quantitative Real Time PCR

Total RNA was extracted with the RNeasy Kit (Qiagen, Hombrechtikon, Switzerland). Following DNase treatment, samples were reverse-transcribed with Oligo (dT) primers using the Omniscript Reverse Transcription Kit (Qiagen). Quantitative reverse transcription-PCR (qRT-PCR) was performed under universal cycling conditions on an ABI 7900HT instrument using commercially available target probes (PAX3, AP2β, FGFR2, FGFR4, CB1, p8 and Bcl-2) and mastermix (all from Applied Biosystems, Rotkreuz, Switzerland). Cycle threshold (*C*_T_) values were normalized to glyceraldehyde-3-phosphate dehydrogenase (GAPDH). Experiments were performed in triplicates. Relative expression levels of the target genes among the different samples were calculated using the ΔΔC_T_ method [22]. Mean values and standard deviations were calculated based on the results of three biological replicates at least.

### Western Blotting

10^7^ Cells were denatured in RIPA buffer supplemented with 1 mM PMSF (Roche) and Roche Complete Protease inhibitor (Roche) for 15 minutes on ice. Total cell extract was separated on 4–12% NuPAGE Bis-Tris gels (Invitrogen, Basel, Switzerland) and blotted on nitrocellulose membranes (Whatman Schleicher & Schuell, Sigma-Aldrich). Blots were blocked with 5% BSA, incubated with the first antibody overnight at 4°C and with the corresponding HRP-conjugated secondary antibody for one hour at RT. Primary antibodies used were: FOXO1 (C20; 1∶500) from Santa Cruz Biotechnology (Heidelberg, Germany), and Caspase 9, recognizing both the pro- and cleaved form, (1∶1000) and PARP (1∶1000) from Cell Signaling (Allschwil, Switzerland). ß-actin antibody (A2103; 1∶2000; Sigma) was used as protein loading control. Enhanced chemiluminescence detection system (SuperSignal West Femto, Pierce, Thermo Scientific, Lausanne, Switzerland) was used for signal detection.

### Apoptosis Assay

Apoptosis was measured after treatment of Rh4 and RMS13 cells with fenretinide for 48 and 96 hrs. Apoptotic index was assessed by flow cytometry, using AnnexinV/FITC (BD, Schwechat, Austria). Data acquisition and analysis were done in a LSR Fortessa Cytometer (BD) using DiVa 6.x software (BD).

### Reactive Oxygen Species (ROS) Generation

Rh4 and RMS13 cells were seeded and treated with fenretinide (IC50) for 24 hours. Afterwards they were incubated for one hour with the profluorescent, lipophilic dye H2-DCF-DA (dihydrodichlorofluorescein diacetate) (Invitrogen) which can diffuse through the cell membrane. H2-DCF-DA final concentration was 20 µM. After reaction with ROS, primarily hydrogen peroxide (H2O2), DCF fluorescence (max. emission ∼ 530 nm) was measured in a microplate reader (Tecan, Männedorf, Switzerland) [23].

### Analysis of miR-9 Expression

RNA was extracted with the miRNeasy kit (Qiagen) following manufactureŕs protocol. Following DNase treatment, samples were reverse-transcribed using the TaqMan MicroRNA Assay Kit (Applied Biosystems). Quantitative reverse transcription-PCR (qRT-PCR) was performed on an ABI 7900HT instrument using commercially available target probe (miR-9) and mastermix (all from Applied Biosystems). Cycle threshold (*C*_T_) values were normalized to snRNA RNU6B. Relative expression levels of miR-9 among the different samples were calculated using the ΔΔC_t_ method [22]. Experiments were performed in triplicates. Mean values and standard deviations were calculated based on the results of two biological replicates.

### In vivo Assay

Xenograft experiments were approved by the veterinary office of the Canton of Zurich. HEK293 GPG cells were transfected with the plasmid pLIB-LN (Takara Bio Europe/Clontech, Saint-Germain-en-Laye, France) expressing *luciferase*. After collection of the retroviral supernatant, Rh4 cells were infected and selection started 48 hours later with G418 (Promega). 3×10^6^ Rh4-*luc* cells were re-suspended in PBS and injected s.c into the flanks of 6 weeks old NOD/Scid Il2rg^−/−^ (NSG) mice (Charles River, Sulzfeld, Germany). Mice bearing tumors were treated intraperitoneally after the tumor reached a volume of at least 100 mm^3^ with either sterile 0.9% NaCl or fenretinide at a dose of 20 mg/kg daily during two weeks. 5 mg fenretinide were dissolved in 106 µl sterile ethanol and then in 1144 µl sterile 0.9% NaCl solution to achieve a final concentration of 4 mg/ml. Tumor growth was measured every day and mice were euthanized when reaching a tumor volume of 1500 mm^3^. Tumor size was determined either by measuring two diameters (d1, d2) in right angles using a digital caliper and volume was calculated using the formula V = (4/3) π r^3^, whereby r = (d1+d2)/4 or by i.p. injection of D-luciferin potassium salt (Caliper Life Sciences, Oftringen, Switzerland), resuspended in sterile aqua ad injectabilia (Sintetica, Mendrisio, Switzerland) to a final concentration of 15 mg/ml, at a dose of 10 µl/g body weight. Tumors were monitored *in vivo* after administration using an IVIS Lumina XR imaging system (Caliper Life Sciences). Total flux (photons/second) was used as the unit of measure. Every treatment group consisted of 3 mice.

### Immunohistochemistry

Mice were sacrificed and tumors obtained by dissection fixed in PFA. Immunohistochemical analysis was done as described before [10]. H&E, Ki67 and cleaved Caspase 3 were stained. For quantitative evaluation, the number of positive cells was counted in ten randomly selected visual fields in non-necrotic areas of the tumor using Image J software. In the case of quantitative analysis of Caspase 3 positive cells, due to the presence of strong staining on the edges of the non treated tumor sections that likely represents an artefact, ten randomly selected visual fields from the inner tumor mass were included. Two-tailed, unpaired t test was used for statistical analysis. The level of significance was set at p<0.05.

### Statistical Analyses

IC50 values were calculated by nonlinear regression curve fitting using GraphPad Prism software (GraphPad Software Inc.). Statistical significance was tested with unpaired two-tailed Student’s *t*-tests and a p<0,05 was considered statistically significant.

## Results

### Fenretinide Modulates PAX3/FOXO1 Target Gene Expression

**Figure 1:**
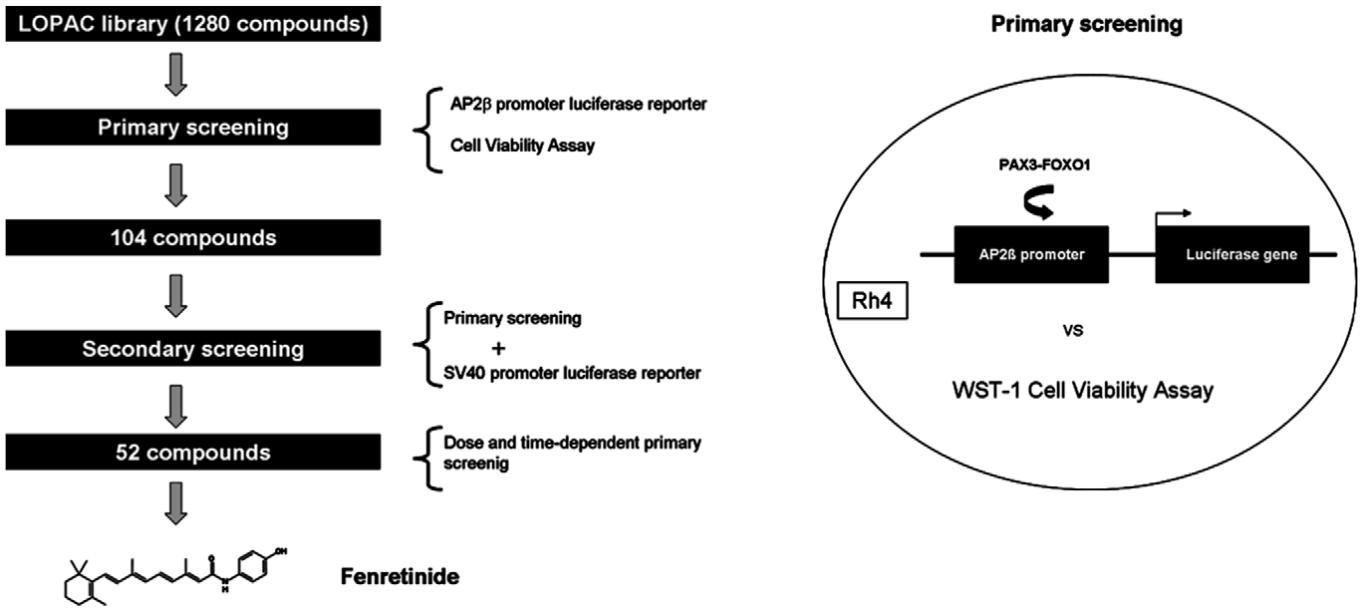
Schematic representation of the screening strategy based on a target gene (AP2β) driven reporter aRMS cell line.

**Figure 2:**
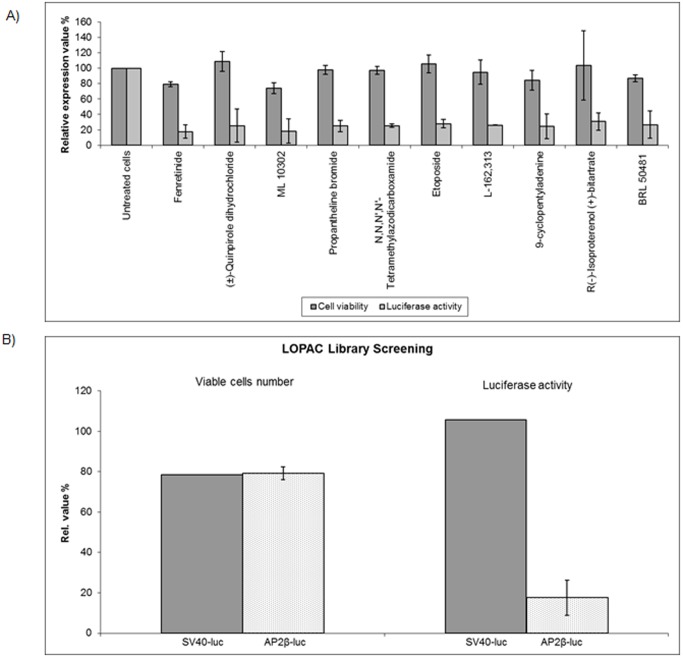
Small-molecule drug screening identifies fenretinide as promising candidate drug. A) Fenretinide inhibits reporter gene activity. Rh4-AP2β-LF cells were treated with the small-molecule compound library at a final concentration of 5 µM during 24 hours and luciferase activity (light grey bars) measured as read-out of the screening together with proliferation (dark grey bars) by WST-1 assay. Actual values for the ten top-ranked compounds are shown relative to untreated controls. B) Proliferation and reporter gene activity in Rh4-AP2β-LF compared to SV40-LF cells after fenretinide treatment for 24 hours are shown. Representative values of three independent experiments. *Columns,* mean; *bars*, s.d.

To identify compounds that might be able to modulate expression of PAX3/FOXO1 target genes either directly or indirectly, we screened a small-molecule compound library (LOPAC 1280, Sigma) which covers 1280 different drug-like and well annotated compounds including all major drug types. We used an endogenous cellular model, Rh4, which represents a PAX3/FOXO1 bearing aRMS cell with a transcription profile very similar to tumor biopsies [24] (screening strategy outlined in Figure 1). As a read-out system we simultaneously assessed cell viability together with a well established and highly sensitive luciferase reporter assay based on the AP2ß target gene promoter to monitor fusion protein activity [10], [11]. For the primary screen, Rh4-AP2ß-LF cells were treated with the compounds at a final concentration of 5 µM for 24 hours. Luciferase activity was measured as primary read-out and corrected for cell number measured in the same wells. Using a cut-off of 65% reduction in luciferase activity, we identified 104 compounds fulfilling all criteria. Among them are molecules with a broad range of mechanisms such as kinases or topoisomerase II inhibitors as well as compounds related to nitric oxide. In a secondary screen, we re-measured all 104 compounds together with a SV40-driven luciferase reporter to eliminate compounds directly affecting luciferase activity. Of the remaining 52 compounds, the top-ranked 10 compounds with the highest ratio proliferation/luciferase activity were then re-tested using different concentrations (5, 1 and 0.5 µM) and time points (24, 48, 72 hours). Fenretinide (retinoic acid p-hydroxyanilide) ranked as the most effective small-molecule compound affecting AP2ß-driven luciferase activity (Table 1 and Figure 2A). Fenretinide-treated cells exerted a reduction in luciferase activity of more than 80% compared to empty vector treated cells whereas proliferation was similar for both cells at this time point (Figure 2B). Hence, fenretinide treatment provoked a non-cytotoxic repression of the PAX3/FOXO1 target gene AP2ß.

**Table 1:** LOPAC small-molecule compound library screening hit list.

Compound	Ratio^a^	Description
Retinoic acid p-hydroxyanilide	4,51	Vitamin A acid analog
(±)-Quinpirole dihydrochloride	4,29	Dopamine receptor agonist
ML 10302	4,04	Serotonin receptor agonist
Propantheline bromide	3,95	Muscarinic acetylcholine receptor antagonist
N,N,N’,N’-Tetramethylazodicarboxamide	3,84	Thiol-oxidizing agent
Etoposide	3,8	Topoisomerase II inhibitor
L-162,313	3,67	Angiotensin II receptor agonist
9-cyclopentyladenine	3,43	Adenylyl cyclase inhibitor
R(−)-Isoproterenol (+)-bitartrate	3,37	Adrenoceptor agonist
BRL 50481	3,27	Phosphodiesterase 7 inhibitor

a Ratio = Proliferation rate/Luciferase activity as compared to the non-treated RH4-AP2ß-LF cells (5 µM incubation for 24 hours).

### Fenretinide Affects aRMS Cell Proliferation at the Low µM Range

To investigate whether fenretinide specifically affects aRMS cells, we next treated four aRMS cell lines (Rh4, Rh41, RMS13, Rh30) with increasing concentrations of fenretinide for 72 hours to determine IC_50_ concentration in comparison to two eRMS cells (RUCH-2 and RD). Fenretinide reduced the number of viable cells in all four aRMS cell lines tested with lower IC_50_ values (IC_50_ range, 2.27–9.42 µM) compared to those of embryonal origin (Ruch-2, RD) that lack PAX3/FOXO1 expression (IC_50_ range, 21.21–31.48 µM) (Table 2). Rh4 was the aRMS cell line most sensitive to fenretinide (2,27 µM*).* As positive control, sensitivity of two ES cell lines, A673 and TC71, was in a very similar range to what has been already reported [25]. Taken together, these results reveal a good sensitivity of translocation-positive RMS cell lines towards fenretinide treatment.

**Table 2:** Fenretinide affects aRMS cell proliferation in the low µM range.

Cell line	Fenretinide IC50^a^	Type
Rh4	2,27 µM	Alveolar
Rh30	9,42 µM	Alveolar
RMS13	6,43 µM	Alveolar
Rh41	5,48 µM	Alveolar
RD	31,48 µM	Embryonal
Ruch-2	21,21 µM	Embryonal
A673	1,09 µM	Ewing Sarcoma
TC71	2,13 µM	Ewing Sarcoma

a IC50 values were calculated by nonlinear regression curve fitting using GraphPad Prism software.

### Fenretinide Reduces Expression of PAX3/FOXO1 mRNA

**Figure 3:**
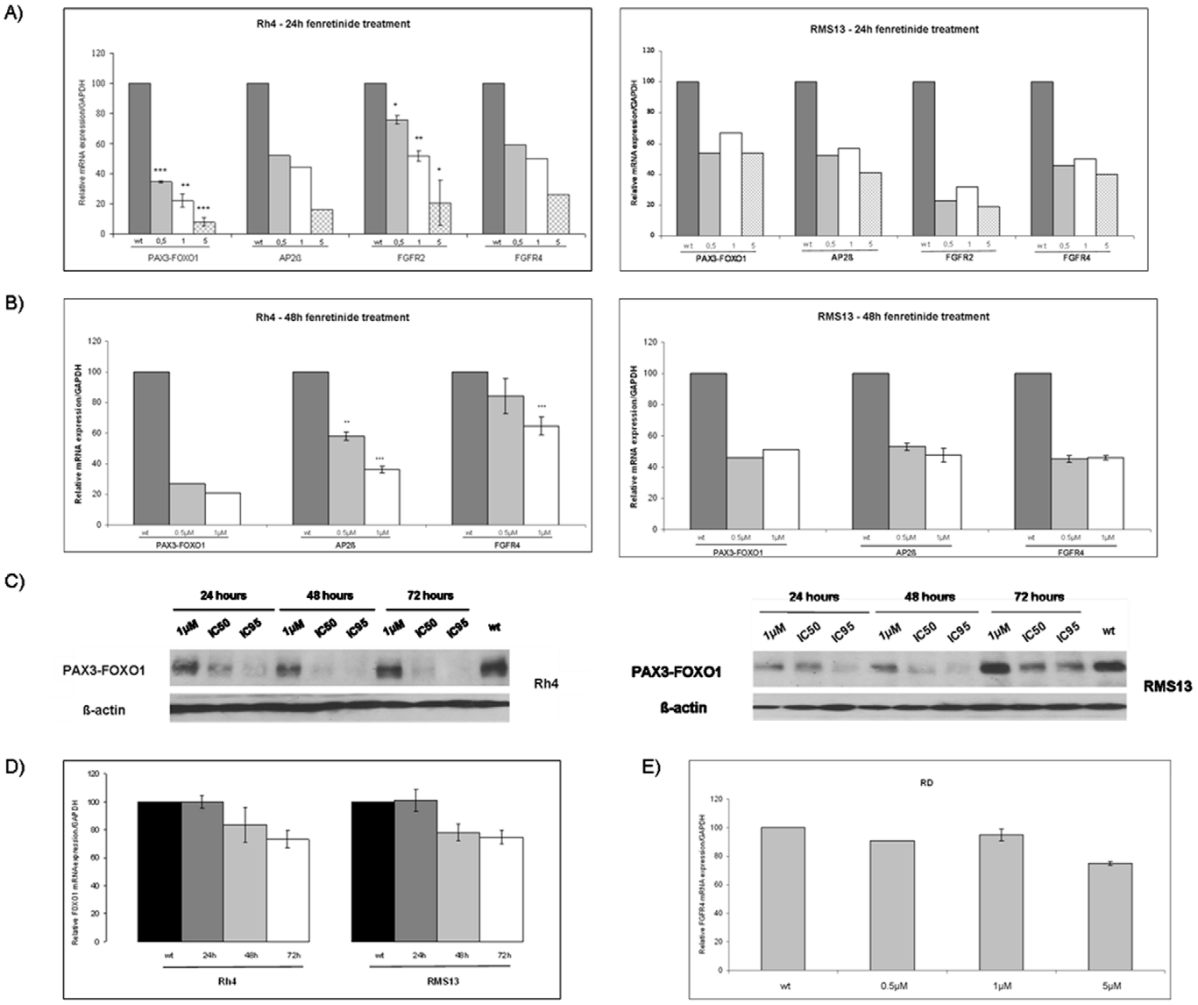
Fenretinide decreases levels of PAX3/FOXO1 and its target genes in aRMS cell lines. Rh4 and RMS13 cells were treated for 24 hours (A) and 48 hours (B) with different concentrations of fenretinide (0.5–1–5 µM, as indicated). PAX3/FOXO1, AP2ß, FGFR2 and FGFR4 mRNA expression levels were measured with qRT–PCR. *C*_T_ values were normalized to GAPDH. Representative values of at least two independently carried out experiments. *Columns,* mean; *bars*, s.d. *** p<0,001 ** p<0,01 * p<0,05 compared to mock treated cells (wt). C) Western blot analysis of PAX3/FOXO1 protein expression in Rh4 and RMS13 cells at different time points of treatment and different doses of fenretinide (1 µM-IC_50_-IC_95_), as indicated. PAX3/FOXO1 was detected with a FOXO1 antibody and actin was used as loading control. D) Fenretinide did not decrease mRNA levels of FOXO1 in aRMS cells. qRT–PCR was carried out after treatment with an IC_50_ concentration of fenretinide during different time points (24–48–72 hours). *C*_T_ values were normalized to GAPDH. Representative values of at least two independent experiments. *Columns,* mean; *bars*, s.d. E) eRMS RD cells were treated during 24 hours with different concentrations of fenretinide (0.5–1–5 µM, as indicated). FGFR4 mRNA expression was measured by qRT-PCR. *C*_T_ values were normalized to GAPDH. Representative values of at least two independently carried out experiments. *Columns,* mean; *bars*, s.d.

To detect early effects of fenretinide on mRNA expression levels of additional PAX3/FOXO1 target genes as well as PAX3/FOXO1 itself, we analyzed mRNA expression by qRT-PCR 24 hours after treatment with different concentrations of the drug (5, 1 and 0.5 µM). Fentretinide led to significant repression of both PAX3/FOXO1 expression and its target genes AP2ß [24], fibroblast growth factor receptor 2 (FGFR2) [24], and fibroblast growth factor receptor 4 (FGFR4) [26], following a dose-dependent course in Rh4 cells. Similar observations were made for RMS13 cells (Figure 3A). Analysis after 48 hours of treatment using lower doses of fenretinide (1 and 0.5 µM) revealed that PAX3/FOXO1 mRNA levels together with its targets AP2ß and FGFR4 were still repressed (Figure 3B). These findings were further corroborated on the protein level in both Rh4 and RMS13 cells (Figure 3C). They suggest that fenretinide treatment affects PAX3/FOXO1 mRNA and protein levels and that of several target genes in translocation-positive RMS cells.

To investigate the specificity of this effect we next measured expression levels of FOXO1 in Rh4 and RMS13 cells. Using an IC_50_ dose of fenretinide, we did not observe any change after 24 hours and only a minor decrease after longer treatment periods (Figure 3D). In addition, the fusion negative eRMS cell line RD was treated for 24 hours with different fenretinide concentrations (5, 1 and 0.5 µM). We did not detect any change in the expression of FGFR4 in RD cells (Figure 3E). These findings suggest that fenretinide affects preferentially PAX3/FOXO1 and its gene expression signature in aRMS.

### Fenretinide Induces Reactive Oxigen Species (ROS) in aRMS

**Figure 4:**
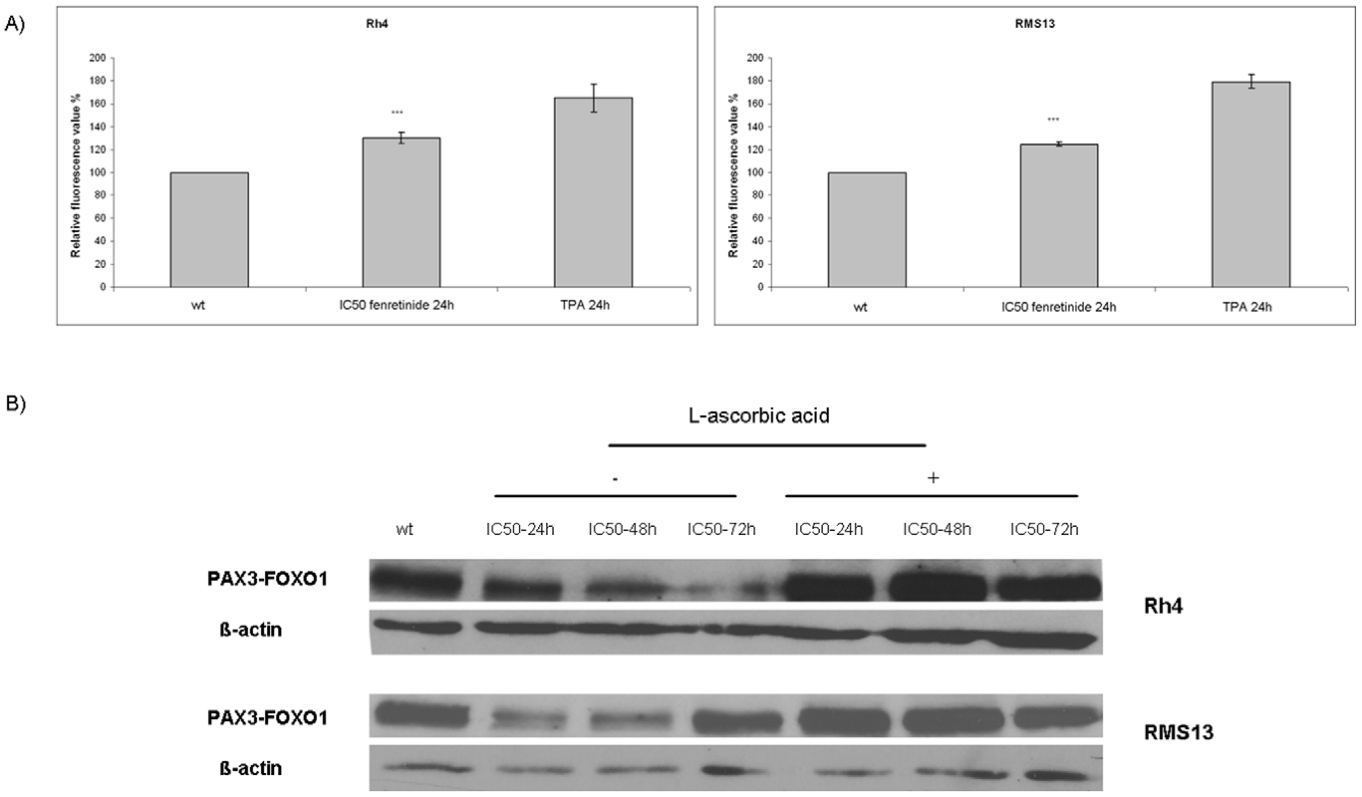
Fenretinide induces ROS in alveolar rhabdomyosarcoma cell lines. A) Rh4 and RMS13 cells were treated for 24 hours with an IC_50_ concentration of fenretinide. H2-DCF-DA was added thereafter for 1 hour. Hydrogen peroxide was added to a final concentration of 250 µM to enhance ROS generation. Final concentration of TPA was 1 µM. Fluorescence was analyzed at 530 nm. The means ± standard deviations (error bars) of three experiments are hown. *Columns,* mean; *bars*, s.d. *** p<0,001 compared to wt. B) The effect induced by fenretinide could be blocked by a ROS inhibitor, L-ascorbic acid. Western blot analysis of Rh4 and RMS13 cells treated with IC_50_ fenretinide alone or a combination of IC_50_ fenretinide plus 100 µM L-ascorbic acid during different time-points (24–48–72 hours). Cells treated with IC_50_ fenretinide plus L-ascorbic acid did not show reduction in PAX3/FOXO1 levels.

To investigate whether fenretinide acts via induction of ROS production in aRMS cells, Rh4 and RMS13 cells were treated with an IC_50_ dose for 24 hours and incubated thereafter with the dye H2-DCF-DA to evaluate intracellular ROS levels. As a positive control for ROS generation, we stimulated cells with TPA [27]. We observed a significant increase of ROS in both Rh4 and RMS13 cells (Figure 4A). Treatment of both aRMS cell lines with L-ascorbic acid, a well-known ROS inhibitor, completely abolished repression of PAX3/FOXO1 protein levels (Figure 4B). These results suggest that fenretinide might affect aRMS cells by the same mechanism that has already been described for other pediatric tumors such as ES and neuroblastoma [25], [28], [29].

### Fenretinide Provokes Apoptosis in aRMS Cells through Caspase 9 Activation and *Bcl-2* Down-regulation

**Figure 5:**
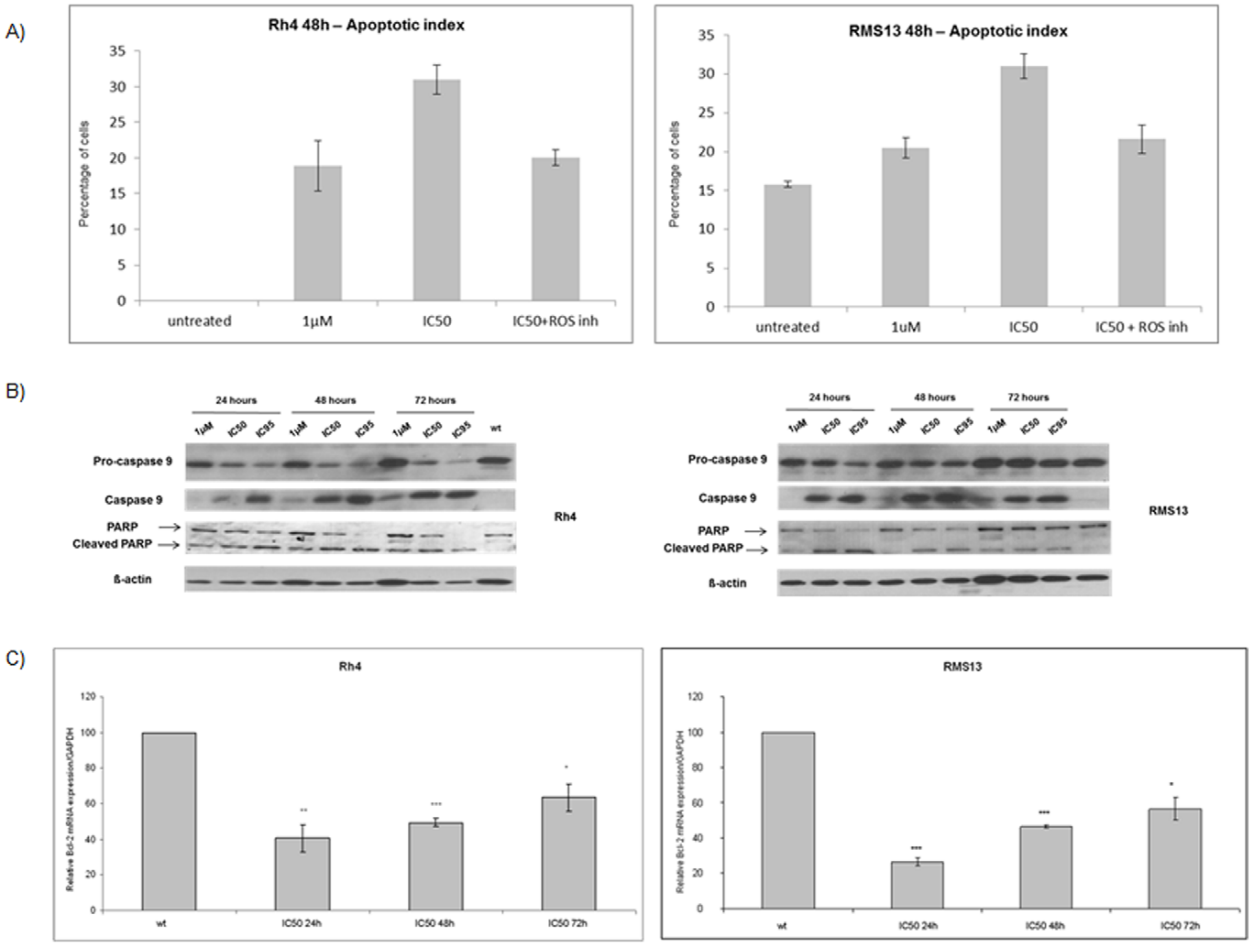
Fenretinide promotes apoptosis in aRMS cells. A) FACS analysis of AnnexinV and PI stained Rh4 and RMS 13 cells after treatment with fenretinide alone (1 µM to IC_50_) or a combination of IC_50_ fenretinide plus 100 µM ascorbic acid (ROS inhibitor) during 48 hours. Graphs show the percentage of apoptotic cells. Representative values of at least two independently carried out experiments. *Columns,* mean; *bars*, s.d. B) Western blot analysis of caspase 9 and PARP cleavage in Rh4 and RMS13 cells after different time points and different doses of fenretinide treatment as indicated. Actin was used as loading control. C) *Bcl-2* mRNA expression levels were determined with qRT-PCR in Rh4 and RMS13 cells treated with IC_50_ concentration of fenretinide at different time points as indicated. *C*_t_ values were normalized to GAPDH. Representative values of three independent experiments. *Columns,* mean; *bars*, s.d. *** p<0,001 ** p<0,01 * p<0,05 compared to wt.

To investigate if the decrease in the number of viable cells after fenretinide treatment was due to apoptosis induction, we performed flow cytometry analysis with combined Annexin V- propidium iodide staining. We observed a dose-dependent increase in the number of cells within the apoptotic fraction (sub-G1) in both aRMS cells. For example in Rh4 cells treated with fenretinide at IC_50_ for 48 hours, the apoptotic index increased to <30% and could be partially blocked by addition of L-ascorbic acid. Similar observations were made with RMS13 cells (Figure 5A). Also, induction of pro-caspase 9 and PARP cleavage in a dose and time-dependent manner was observed (Figure 5B). Next, we assessed the mRNA expression levels of the anti-apoptotic protein Bcl-2 by qRT–PCR. Fenretinide prompted a marked decrease in Bcl-2 levels particularly after 24 hour exposure in both aRMS cell lines (Figure 5C). Collectively, these results suggest that fenretinide induces apoptosis in aRMS cells via caspase 9 activation and a marked decrease in Bcl-2 levels.

### Fenretinide Induces miR-9 Expression

**Figure 6:**
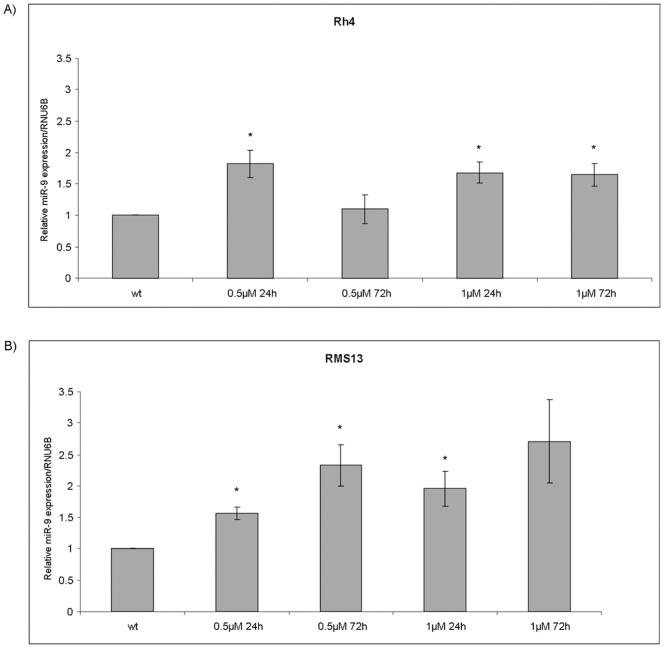
Fenretinide upregulates miR-9. Rh4 and RMS13 cells were treated with different concentrations of fenretinide at two time points (0.5–1 µM; 24–72 hours). miR-9 expression level was measured by qRT-PCR. *C*_T_ values were normalized to snRNA RNU6B. Representative values of three independently carried out experiments. *Columns,* mean; *bars*, s.d. * p<0,05 compared to wt.

It has been shown previously that fenretinide is able to increase miR-9 levels in human retinal pigment epithelial cells [30]. Hence, we analyzed expression of miR-9 after two low dose (0.5 and 1 µM) treatment at two different time points (24 and 72 hours) by qRT-PCR. Significant up-regulation of miR-9 was seen in both aRMS cell lines (Figure 6A, B).

### Fenretinide Delays Tumor Growth *in vivo*

**Figure 7:**
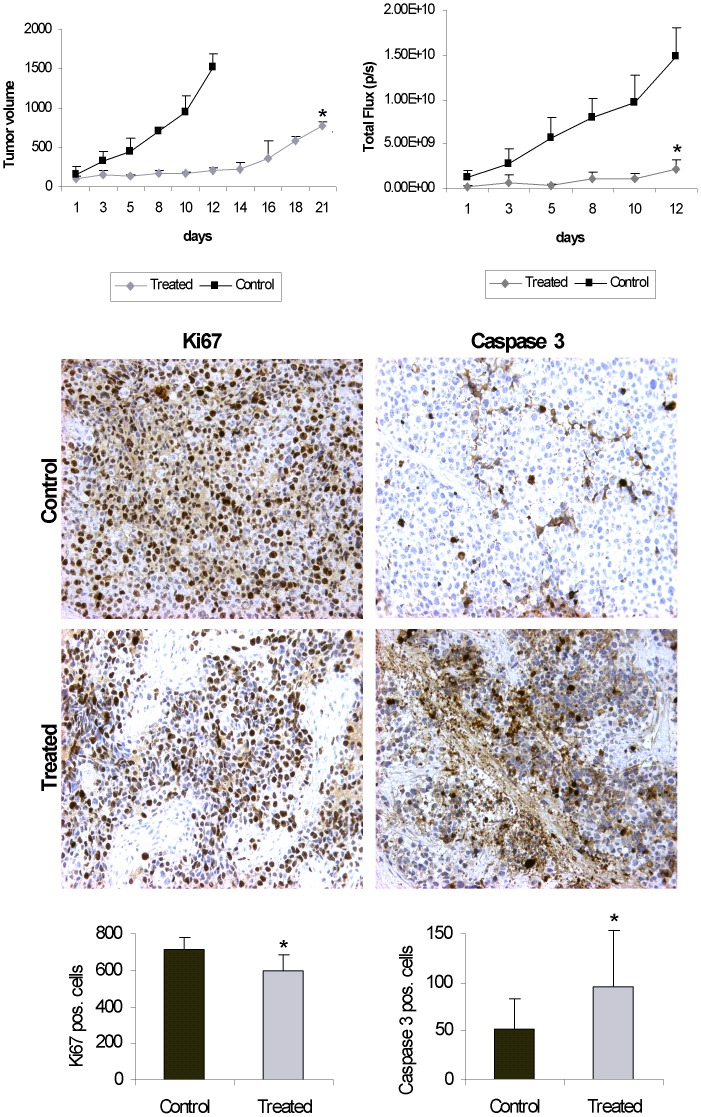
Fenretinide delays tumor growth *in vivo*. Growth rate of Rh4 xenograft tumors in NOD/Scidil2rg^−/−^ mice (n = 3) treated with sterile 0.9% NaCl (control) or fenretinide (treated), Left: tumor volume was determined using a calliper, Right: tumor volumes determined according to luminescence values measured by IVIS Lumina XR imaging system (Caliper Life Sciences). B) Immunohistological staining of Ki67 (left) and activated caspase 3 (right) of Rh4 xenograft tumor sections treated with sterile 0.9% NaCl or fenretinide. C) Quantification of Ki67 and activated caspase 3 positive cells counted per field using Image J software in Rh4 sections of tumors treated with sterile 0.9% NaCl or fenretinide. *Columns,* mean; *bars*, s.d. *p<0,05 compared to control mice.

Finally, we used an aRMS xenograft mouse model generated by subcutaneous injection of Rh4 cells engineered to constitutively express luciferase into immunocompromised NOD/Scidil2rg^−/−^ mice to analyze the effects of fenretinide *in vivo*. Treatment with fenretinide at a dose of 20 mg/kg daily during two weeks significantly slowed down tumor growth compared to control mice both when measuring tumor volume (Figure 7A, left) as well as luciferase activity (Figure 7B, right). To further characterize the effect of fenretinide, tumors were isolated, paraffin-embedded and immunohistochemically stained with the proliferation marker Ki67 and activated caspase 3. As shown in Figure 7B, the number of both Ki-67 and caspase 3 positive cells was clearly reduced in the treated tumors, with large areas without positive cells. Quantitative analysis revealed that the reduction was statistically significant. We did also not observe any adverse physiological effects in the treated mice. Hence, fenretinide displays an anti-tumorigenic effect also *in vivo*.

## Discussion and Conclusion

aRMS is in urgent need of new therapeutic approaches as resistance to current chemotherapeutic regimens is unfortunately common [31] and relapsed and metastatic patients show a very poor prognosis [13], [32]. In this study, we have used a library of 1280 well characterized small-molecule compounds together with an endogenous aRMS cellular model (Rh4) to screen for the most active compounds. We have identified fenretinide as the most effective agent reducing PAX3-FOXO1 levels *in vitro* and decreasing tumor growth *in vivo*. While most of the fenretinide-treated cells remained alive after short exposure, they already displayed a marked reduction in AP2ß-driven luciferase activity (up to 80% decrease) as compared to non-treated cells. Even more striking, fenretinide treatment resulted in a significant reduction of PAX3-FOXO1 mRNA and protein levels, and consequently affected expression of its target genes. To our knowledge, this is the first report of fenretinide activity in aRMS.

Fenretinide (N-(4-hydroxyphenyl)retinamide) is a synthetic retinoid derivative that holds potential both as anticancer and chemopreventive drug. It is well tolerated both in adult and paediatric patients [33]. Moreover, the drug demonstrated activity both *in vitro* and *in vivo* against a wide range of tumors [34], [35] including childhood neuroblastoma and ES [36], [37]. Hence, several clinical trials with fenretinide in paediatric cancer patients are ongoing [18], [38]. Retinoic acid (RA) and derivatives such as ATRA (all-trans-retinoic acid) and CRA (13-cis retinoic acid) have demonstrated effects inhibiting growth and promoting differentiation of some, but not all RMS cell lines [39], [40]. We have shown that fenretinide was able to inhibit the growth of aRMS cells at low µM concentration (Rh4 IC_50_ 2,27 µM and RMS13 IC_50_ 6 µM). aRMS cells were more sensitive to fenretinide than translocation-negative eRMS cell lines. These IC_50_ values are very similar to the ones already described in other paediatric neoplasias such as rhabdoid tumors [41] and only slightly higher than in ES cells [25].

Fenretinide alters the PAX3/FOXO1 dependent transcriptional program since it represses PAX3/FOXO1 at both mRNA and protein levels. Consequently, also well-defined targets such as AP2ß, FGFR2 or FGFR4 [24], [26] are repressed. Among these, FGFR4 is known to play an important role in aRMS since activating mutations in its tyrosine kinase domain have been reported in a subset of primary tumors associated with an enhanced metastatic phenotype in xenografts [42]. AP2ß has been validated as direct target gene mediating the anti-apoptotic and proliferative function of PAX3/FOXO1 and FGFR2 is part of the PAX3/FOXO1 *in vivo* target gene signature [24]. In Rh4 and RMS13 cells levels of FGFR4; AP2ß and FGFR2 are diminished by fenretinide using lower doses such as 0.5 and 1 µM and they remained repressed for 72 hours of treatment. Fenretinide also reduced protein levels of PAX3/FOXO1 in both cell lines and as expected, FGFR4 protein levels were also found to be repressed (data not shown). Fenretinide treatment also down-regulated CB1 (data not shown), another direct target gene of PAX3/FOXO1 [6], confirming an inverse correlation between these two genes that was reported previously [43]. Fenretinide modified FOXO1 levels only minor in aRMS cells and *FGFR4* expression was not changed in eRMS cell line RD that lacks the fusion protein. Thus, fenretinide affected PAX3/FOXO1 gene expression signature preferentially in translocation-positive RMS cells as these effects can not be observed in translocation-negative RMS cell lines. We conclude that fenretinide reduces PAX3/FOXO1 levels and most importantly PAX3/FOXO1 transcriptional activity, at least at the level of target gene expression. However, the mechanisms leading to reduction in PAX3/FOXO1 mRNA levels remain to be investigated further. Since very little is known about transcriptional regulation of PAX3/FOXO1 in aRMS cells, identification of fenretinide provides a first important tool the address this issue in the future.

Fenretinide induced apoptosis in both aRMS cell lines similar to what has already been described in many other tumors [17]. Modulation of expression levels of anti-apoptotic genes such as Bcl-2 has been described in leukaemia cells exposed to fenretinide [44]. Bcl-2 expression is of significance in rhabdomyosarcoma as high levels have been linked to poor survival and recurrence [45]. Fenretinide is able to down-regulate Bcl-2 levels in both cell lines. Fenretinide treatment induced pro-caspase 9 cleavage as well as caspase 3/7 activation (data not shown) in a dose and time-dependent manner in both Rh4 and RMS13 cells. Corresponding PARP cleavage was also observed (data not shown). Caspase 9 activation due to fenretinide treatment confirms the initiation of the intrinsic or mitochondrial-mediated apoptotic pathway as has been described in ES [28]. This might possibly be mediated by generation of ROS as implicated also in other tumor cell types analyzed and described in detail before [17] in cells including ES [25], [28]. Indeed, we could also observe increased ROS production in aRMS cells. Processing of pro-caspase 9 and the down-regulation of Bcl-2 and AP2ß could explain the pro-apoptotic effect of fenretinide in aRMS cells. However, the exact mechanism by which production of ROS might be linked to repression of PAX3/FOXO1 and Bcl-2 need to be further investigated. When aRMS cells were treated with L-ascorbic acid, a well-known ROS inhibitor, fenretinide effects were completely abolished.

MicroRNAs (miRs) are small highly conserved non-coding RNAs [46]. Deregulation of miRs expression is a common phenomena in cancer [47] and they have emerged as promising therapeutic targets [48]. miR-9 has been described both as tumor suppressor [49], [50], and as an oncomiR involved in metastasis formation [51]. A link between miR-9 and stem cells have been reported [52], [53]. Importantly, miR-9 has been connected to ROS generation [54], and an increase in miR-9 expression levels has been described in human retinal epithelial cells after fenretinide exposure [30]. Here, we could demonstrate that fenretinide is able to increase miR-9 expression in aRMS as well. So far this is the first description of miR-9 expression in aRMS and its possible function in aRMS metastasis warrants further investigation.

*In vivo,* fenretinide delayed tumor growth and metabolic rate in an Rh4 mouse xenograft model. Effectiveness of fenretinide *per se* has been demonstrated *in vivo* in a wide range of tumors such as ovarian cancer [55], Kaposi’s sarcoma [56], retinoblastoma [57] but also in combination with other chemotherapeutic agents as rituximab in B-cell lymphoma [58], ABT-737 in neuroblastoma [59] or genistein in ES xenografts [60]. Synergy of fenretinde with ABT-737 in acute lymphoblastic leukemia cell lines depends on Mcl-1 inactivation [61] and in neuroblastoma on Bcl-2 inhibition [59]. The synergistic induction of apoptosis observed in ES with fenretinide and death receptors ligands suggests another exciting possibility [62]. Since stimulation of death receptors is also able to induce apoptosis in aRMS, combination with fenretinide might be indeed an interesting possibility to further improve its anti-tumorigenic effects [63]. Hence, it will be a goal of future research to identify drug combinations that act synergistically with fenretinide in aRMS, especially in larger *in vivo* studies.

Here, we have demonstrated that fenretinide is effective *in vitro* and *in vivo* against aRMS and warrants further investigation especially in combination with other drugs. Unlike most other experimental strategies which reason that an increased understanding of the pathobiology (target genes) would lead to identification of active compounds, our strategy was based on a reverse approach that has allowed us to identify already known compounds which have not yet been tested for aRMS treatment, such as fenretinide.
